# Low Detection Rates of Genetic FH in Cohort of Patients With Severe Hypercholesterolemia in the United Arabic Emirates

**DOI:** 10.3389/fgene.2021.809256

**Published:** 2022-01-03

**Authors:** Antoine Rimbert, Hinda Daggag, Peter Lansberg, Adam Buckley, Martijn Viel, Roan Kanninga, Lennart Johansson, Robin P. F. Dullaart, Richard Sinke, Alia Al Tikriti, Jan Albert Kuivenhoven, Maha Taysir Barakat

**Affiliations:** ^1^ Department of Paediatrics, Section Molecular Genetics, University Medical Centre Groningen, University of Groningen, Groningen, Netherlands; ^2^ Imperial College London Diabetes Centre, Abu Dhabi, United Arab Emirates; ^3^ Department of Genetics, University Medical Centre Groningen, University of Groningen, Groningen, Netherlands; ^4^ Department of Endocrinology, University Medical Centre Groningen, University of Groningen, Groningen, Netherlands

**Keywords:** familial hypercholestelemia, genetics, screening, prevalence, United Arab Emirates (UAE)

## Abstract

**Background:** Programs to screen for Familial hypercholesterolemia (FH) are conducted worldwide. In Western societies, these programs have been shown to be cost-effective with hit/detection rates of 1 in 217–250. Thus far, there is no published data on genetic FH in the Gulf region. Using United Arab Emirates as a proxy for the Gulf region, we assessed the prevalence of genetically confirmed FH in the Emirati population sample.

**Materials and Methods:** We recruited 229 patients with LDL-C >95^th^ percentile and employed a customized next generation sequencing pipeline to screen canonical FH genes (*LDLR, APOB, PCSK9, LDLRAP1*).

**Results:** Participants were characterized by mean total cholesterol and low-density lipoprotein cholesterol (LDL-c) of 6.3 ± 1.1 and 4.7 ± 1.1 mmol/L respectively. Ninety-six percent of the participants were using lipid-lowering medication with mean corrected LDL-c values of 10.0 ± 3.0 mmol/L 15 out of 229 participants were found to suffer from genetically confirmed FH. Carriers of causal genetic variants for FH had higher on-treatment LDL-c compared to those without causal variants (5.7 ± 1.5 vs 4.7 ± 1.0; *p* = 3.7E-04). The groups did not differ regarding high-density lipoprotein cholesterol, triglycerides, body mass index, blood pressure, glucose, and glycated haemoglobin.

**Conclusion:** This study reveals a low 7% prevalence of genetic FH in Emiratis with marked hypercholesterolemia as determined by correcting LDL-c for the use of lipid-lowering treatment. The portfolio of mutations identified is, to a large extent, unique and includes gene duplications. Our findings warrant further studies into origins of hypercholesterolemia in these patients. This is further supported by the fact that these patients are also characterized by high prevalence of type 2 diabetes (42% in the current study cohort) which already puts them at an increased risk of atherosclerotic cardiovascular disease. These results may also be useful in public health initiatives for FH cascade screening programs in the UAE and maybe the Gulf region.

## Introduction

Atherosclerotic cardiovascular disease (ASCVD) is the leading cause of death in the Middle East (Bamimore et al., 2015). Hypercholesterolemia, characterised by increased levels of low-density lipoprotein cholesterol (LDL-c), is a major established risk factor for ASCVD (Nordestgaard et al., 2013; Ference et al., 2017). It is well-established that functional mutations in *LDLR* (Brown and Goldstein, 1974), *APOB* (Innerarity et al., 1987) and *PCSK9* (Abifadel et al., 2003) can cause autosomal dominant hypercholesterolemia and that mutations in *LDLRAP1* (Ceska et al., 2019) can cause autosomal recessive hypercholesterolemia. Combined, this set of genetic disorders is generally referred to as familial hypercholesterolemia (FH). Since patients with FH are characterized by a 22-fold increased risk for ASCVD, compared to non-carriers with comparable LDL-c concentrations (Abul-Husn et al., 2016; Khera et al., 2016), knowing the aetiology of severe hypercholesterolemia is of major importance for an early diagnosis and pro-active cardiovascular healthcare.

Programs to identify patients with FH are conducted worldwide to improve management and enable early prevention through cascade screening (Vallejo-Vaz et al., 2018; Ceska et al., 2019). These initiatives (reviewed in (Knowles et al., 2017)) have been shown to reduce the average age at which individuals with genetic FH are diagnosed, to improve treatment initiation/adherence (Perez de Isla et al., 2016), and reduce LDL-c and ASCVD in a cost-effective way (Wonderling et al., 2004; Nherera et al., 2011).

The prevalence of clinically defined FH is currently estimated to be 1:217–250 across different Caucasian populations (Benn et al., 2016; de Ferranti et al., 2016). In patients who are referred to specialized lipid clinics, the proportion of genetically confirmed cases can be 50% or more depending on the inclusion criteria (Wang et al., 2016).

A reportedly high prevalence of clinical FH in the Arabian Gulf region (Al-Rasadi et al., 2018a) recently increased awareness of severe dyslipidaemias and a need for cascade screening. Several promising large-scale initiatives are currently under way, but to date publicly available data on a genetic diagnosis of FH in this part of the world are very limited (Bamimore et al., 2015; Al-Rasadi et al., 2018a).

In this study, we screened patients that were referred to the Imperial College London Diabetes Centre in Abu Dhabi (ICLDC), for LDL-C above the 95% percentile. Ninety-six percent of the patients were using lipid-lowering medication while 42% were suffering from type 2 diabetes. The mean LDL-C value corrected for lipid-lowering medication was 10 mmol/L but we could only identify a genetic origin for FH in 7% of the patients.

## Material and Methods

### Participants

229 unrelated Emiratis were recruited using the LDL-C cut-off levels used in the Gulf FH Registry final protocol conditions (https://gulfheart.org) [(Al-Rasadi et al., 2018a; Al-Rasadi et al., 2018b) and Supplemental methods]. In brief, adult patients were recruited by Imperial College London Diabetes Centre in Abu Dhabi (ICLDC), presenting with plasma LDL-c concentrations higher than 4.9 mmol/L (with or without lipid lowering treatment) and plasma triglycerides <5 mmol/L. Patients suffering from history of untreated hypothyroidism; history of proteinuria ≥1g/L; history of obstructive liver disease; history of chronic renal failure; human immunodeficiency virus infection or on immunosuppressant or steroid or psychiatric medications, were excluded. Data on family history and physical examination were not available. Ethical approval was obtained from ICLDC Research Ethics Committee. Participants were consented for research by a research patient recruitment officer and were asked for additional blood samples during their regular clinical visit to ICLDC.

### Molecular Genetics Analysis

All samples were analysed for autosomal dominant or autosomal recessive hypercholesterolemia (*LDLR*, *APOB*, *PCSK9*, *LDLRAP1*) using a custom targeted next generation sequencing gene panel. The analysis pipeline focused on: 1- rare genetic variants in the general population [including a control population from ME countries (Scott et al., 2016)]; 2- genetic variants affecting coding and splicing regions of targeted genes or located in promoter regions of *LDLR*. Pathogenicity of identified variants was determined using clinical genetic databases and *in silico* prediction algorithms (see Supplemental Methods). The targeted genes were tested for copy number variations (CNV) using the same analysis pipeline (Johansson et al., 2016; Balder et al., 2018) and validated using the Infinium Global Screening Array (Illumina®). Full details of the technical approach are provided in the supplementary methods.

### Statistics

T-test was used to compare the lipid parameters between carriers of causal variants in *LDLR, APOB* and *PCSK9* and non-carriers and Chi^2^ test was used to compare the proportion of participants between subgroups.

## Results

### Characteristics of Study Participants

Baseline characteristics of the 229 participants (118 women and 111 men) are shown in Table 1. Mean age of the participants at consent was 46 years (±10). Patients presented with average body mass index of 31 (±6). On treatment plasma lipids and lipoproteins were as follows: Total cholesterol 6.3 mmol/L (±1.1), LDL-c: 4.7 mmol/L (±1.1), High-density lipoprotein cholesterol (HDL-c): 1.3 mmol/L (±0.3); Triglycerides: 2.0 mmol/L (±0.9). Ninety-six percent of the patients were using lipid-lowering drugs (Rosuvastatin (*n* = 106), Atorvastatin (*n* = 71), Pitavastatin (*n* = 6); Simvastatin (*n* = 2), Rosuvastatin+Ezetimibe (*n* = 22), Atorvastatin+Ezetimibe (*n* = 10), Simvastatin+Ezetimibe (*n* = 2); and Ezetimibe (*n* = 1). When corrected for the reported use of lipid-lowering drugs (conversion factors are given in Supplementary Table S1), the corrected LDL-c was on average 10 mmol/L (±3.0).

**TABLE 1:** Baseline parameters of 229 participants with a diagnosis of clinical FH.

	All (n = 229)
Sex	118 W, 111 M
Age at consent (years), [Mean, (±SD)]	46 (±10)
Plasma lipids	
Total Cholesterol (mmol/L), [Mean, (±SD)]	6.3 (±1.1)
LDL cholesterol (mmol/L), [Mean, (±SD)]	4.7 (±1.1)
HDL cholesterol (mmol/L), [Mean, (±SD)]	1.3 (±0.3)
Triglycerides (mmol/L), [Mean, (±SD)]	2.0 (±0.9)
Corrected LDL cholesterol (mmol/L), [Mean, (±SD)]	10.0 (±3.0)
Lipid lowering drugs^$^ [n, (%)]	219 (96%)
Anthropometric data	
BMI (kg/m2), [Mean, (±SD)]	31 (±6)
BP Systolic (mmHg), [Mean, (±SD)]	125 (±17)
BP Diastolic (mmHg), [Mean, (±SD)]	74 (±10)
Glucose (mmol/L), [Mean, (±SD)]	8.3 (±4.2)
HbA1c (%), [Mean, (±SD)]	7.2 (±2.1)
Diabetes status	
Non diabetes [*n*, (%)]	81 (35%)
Pre-diabetes [*n*, (%)]	48 (21%)
Type 2 Diabetes Mellitus [*n*, (%)]	96 (42%)
Type 1 Diabetes [*n*, (%)]	4 (2%)

Legend and abbreviations: LDL-c, low-density lipoprotein cholesterol; HDL, high-density lipoprotein cholesterol; LDL-c plasma levels were calculated using Friedewald’s formula (Friedewald et al., 1972); SD, standard deviation; BMI, body mass index; $, lipid-lowering medication includes atorvastatin, Rosuvastatin or Simvastatin and/or Ezetimibe; Correction factors can be found in Supplementary Table S1; BP, blood pressure; HbA1c, glycated haemoglobin; On the basis of current ADA criteria (American Diabetes Associa, 2018), patients presenting HbA1c<5.7% were considered as non-diabetes, 5.7 ≤ HbA1c ≤ 6.4% as pre-diabetes and HbA1c>6.4% as type 2 diabetes mellitus.

### Genetics of Hypercholesterolemia

Targeted sequencing rendered a mean coverage depth of 678X (±263) for each base with 98% (±0.01) of the targeted regions covered at least 30 times. This result allowed for an efficient and robust detection of heterozygous variants as well as copy number variations calling.

### Prevalence of Genetic FH

Fifteen out of 229 participants (7%) were diagnosed with genetically defined FH (FH^+^). Thirteen variants were located in coding (missense or frameshift mutations) or promoter regions of *LDLR* gene; one in *APOB* and one in *PCSK9* (Table 2). Two rare causal variants were identified only twice which does not support a hypothesis of large impact of founder mutations in Emiratis, as is the case in the Lebanese population (Abifadel et al., 2009). We did not observe known causal rare variants in the *APOB* gene which in Western countries accounts for 13% of the FH cases (Abul-Husn et al., 2016; Khera et al., 2016) but we did identify 28 rare *APOB* variants of unknown clinical significance (Supplementary Table S2). Follow-up studies are needed to better understand and determine the potential causality of these variants.

**TABLE 2:** FH mutations identified in 229 participants with clinical FH.

Patients IDs	Chrom_pos_ref_alt	Gene Symbol	Coding	Protein	Automatic pathogenic	ClinVar	Predicted Damaging	Publishedevidences
53	Chr19_11216114_G_A	*LDLR*	c.532G > A	p.Asp178Asn			Yes	
	Chr19_11217274_G_A	*LDLR*	c.728G > A	p.Cys243Tyr			Yes	
99	Chr19_11224051_C_G	*LDLR*	c.1284C > G	p.Asn428Lys			Yes	
218	Chr19_11224281_G_A	*LDLR*	c.1429G > A	p.Asp477Asn			Yes	
352	Chr19_11200090_C_G	*LDLR*	c.-135C > G			Yes		Yes
391	Chr19_11221366_C_T	*LDLR*	c.979C > T	p.His327Tyr			Yes	
423	Chr1_55509631_T_G	*PCSK9*	c.323T > G	p.Leu108Arg		Yes		
	Chr19_11224281_G_A	*LDLR*	c.1429G > A	p.Asp477Asn			Yes	
622	Chr19_11224061_C_G	*LDLR*	c.1294C > G	p.Leu432Val			Yes	
634	Chr19_11226789_TG_T	*LDLR*	c.1610delG	p.Gly537fs	Yes			
662	Chr2_21224256–21286077_Dup	*APOB*			Yes			
689	Chr19_11224407_C_T	*LDLR*	c.1555C > T	p.Pro519Ser		Yes		
720	Chr19_11216085_ACAACGAC_A	*LDLR*	c.505_511delAACGACC	p.Asn169fs	Yes			
750	Chr19_11224233_G_A	*LDLR*	c.1381G > A	p.Gly461Ser				Yes
808	Chr19_11224407_C_T	*LDLR*	c.1555C > T	p.Pro519Ser		Yes		
813	Chr19_11200105_C_T	*LDLR*	c.-120C > T			Yes		Yes
851	Chr19_11215960_C_A	*LDLR*	c.378C > A	p.Phe126Leu			Yes	

Legend and abbreviations: Genomic coordinates of identified variants are reported with Chromosome, Position, Reference Allele and Alternative Allele observed (Chrom_Pos_Ref_Alt) related to GRCh37 genome Human built 19*.* All variants listed are heterozygous*.* LDLR, low density lipoprotein receptor (NM_000527); *PCSK9*, Proprotein convertase subtilisin/kexin type 9 (NM_174936.3); UTR, untranslated regions; LDL-c, low density lipoprotein cholesterol, M, males; F, females; dup, duplication. LDL-c plasma levels were corrected using the Friedewald’s formula (Friedewald et al., 1972). *LDLR*, p.Cys243Tyr, p.Asn169fs and p.Gly537fs and *APOB_*dup are reported for the first time.

### Novel Mutations

Of the mutations identified, none were previously reported in the Middle East so far (Bamimore et al., 2015). Among the fifteen identified causative FH mutations, *LDLR* p. Asn169fs, p. Cys243Tyr and p. Gly537fs are reported for the first time worldwide according to current publicly available information.

### Compound Heterozygous FH

Two participants were identified as compound heterozygotes. The first case (Table 2; ID:#53), a 41 years old woman with LDL-c of 9.4 mmol/L on lipid-lowering treatment (Simvastatin 20 mg + Ezetimibe 10 mg) (corrected LDL-c = 18.8 mmol/L), carried two mutations in *LDLR* (p.Asp178Asn and p. Cys243Tyr). The genetic analysis performed does not allow for determining if these mutations are located on the same allele. The second case (Table 2; ID:#423), a 45 years old man with LDL-c of 6.8 mmol/L on lipid-lowering treatment (Rosuvastatin 20 mg + Ezetimibe 10 mg) (corrected LDL-c = 18.2 mmol/L), carried a variant in *LDLR* (p.Asp477Asn) and one in *PCSK9* (p.Leu108Arg), both previously reported as FH causal mutations.

### Duplication of APOB

Here we report a duplication of the entire gene *APOB* in a 29 year old man (Table 2; ID:#662) with LDL-c of 6.5 mmol/L under lipid-lowering treatment (Rosuvastatin 20 mg; corrected LDL-c = 17.5 mmol/L). This ∼1.2 Mb long duplication [chr2: 21,130,084–22,324,616 (Hg19)] has been validated using Global Screening Array (Supplemental Methods and Supplementary Figure S1). Whether 3 *APOB* gene copies can cause FH is topic of on-going studies, but this specific case illustrates the potential of next generation sequencing to improve our understanding of previously unexplained FH.

### FH^-^ Versus FH^+^

Compared to FH^-^, the FH^+^ group had significantly higher total cholesterol (7.2 vs. 6.2 mmol/L; *p* = 2.1e-03), on treatment LDL-c (5.7 vs 4.7 mmol/L; *p* = 3.7e-04), and corrected LDL-c (9.8 mmol/L (±2.7) vs. 13.9 mmol/L (±4.5); *p* = 2.1E-07). The FH^−^ and FH^+^ group were similar with regard to gender distribution, age, BMI, blood pressure, plasma glucose concentration, HbA1c, HDL-c, and triglycerides plasma levels (Table 3).

**TABLE 3:** Differences between participants with (FH^+^) and without (FH^-^) rare causal variants in *LDLR*, *APOB*, and *PCSK9*.

	FH^-^ (n = 214)	FH^+^ (n = 15)	P value
Sex	112 W, 102 M	6 W, 9 M	0.51
Age at consent (years), [Mean, (±SD)]	46 (±10)	41 (±11)	0.08
Plasma lipids			
Total Cholesterol (mmol/L), [Mean, (±SD)]	6.2 (±1.1)	7.2 (±1.8)	0.0021
LDL cholesterol (mmol/L), [Mean, (±SD)]	4.7 (±1.0)	5.7 (±1.5)	0.00037
HDL cholesterol (mmol/L), [Mean, (±SD)]	1.3 (±0.3)	1.2 (±0.3)	0.28
Triglycerides (mmol/L), [Mean, (±SD)]	2.0 (±0.9)	1.7 (±0.7)	0.18
Corrected LDL cholesterol (mmol/L), [Mean, (±SD)]	9.8 (±2.7)	13.9 (±4.5)	0.00000021
Lipid lowering drugs^$^ [*n*, (%)]	204 (95%)	15 (100%)	NA
Anthropometric data			
BMI (kg/m2), [Mean, (±SD)]	31 (±6)	30 (±4)	0.46
BP Systolic (mmHg), [Mean, (±SD)]	125 (±17)	120 (±13)	0.23
BP Diastolic (mmHg), [Mean, (±SD)]	74 (±10)	72 (±7)	0.48
Glucose (mmol/L), [Mean, (±SD)]	8.4 (±4.2)	7.2 (±4.1)	0.29
HbA1c (%), [Mean, (±SD)]	7.2 (±2.1)	6.6 (±2.0)	0.36
Diabetes repartition			
Non diabetes [*n*, (%)]	74 (35%)	7 (47%)	0.34
Pre-diabetes [*n*, (%)]	45 (21%)	3 (20%)	0.92
Type 2 Diabetes Mellitus [*n*, (%)]	91 (42%)	5 (33%)	0.49
Type 1 Diabetes [*n*, (%)]	4 (2%)	0 (0%)	NA

Legend and abbreviations: FH, familial hypercholesterolemia; FH^-^ non genetically defined FH, FH^+^, genetically defined FH; SD, standard deviation; BMI, body mass index; LDL-c, low-density lipoprotein cholesterol; HDL, high density lipoprotein cholesterol; $: lipid-lowering medication includes atorvastatin, rosuvastatin or simvastatin and/or Ezetimibe. Correction factors can be found in Supplementary Table S1. LDL-c plasma levels were calculated using the Friedewald’s formula (Friedewald et al., 1972). NA, non applicable. T-test was used to compare the lipid parameters between FH mutation carriers and non-carriers and Chi2 test was used to compare the proportion of participants between groups.

### Percentages of Individuals With FH Within Different LDL-C Ranges

**FIGURE 1:**
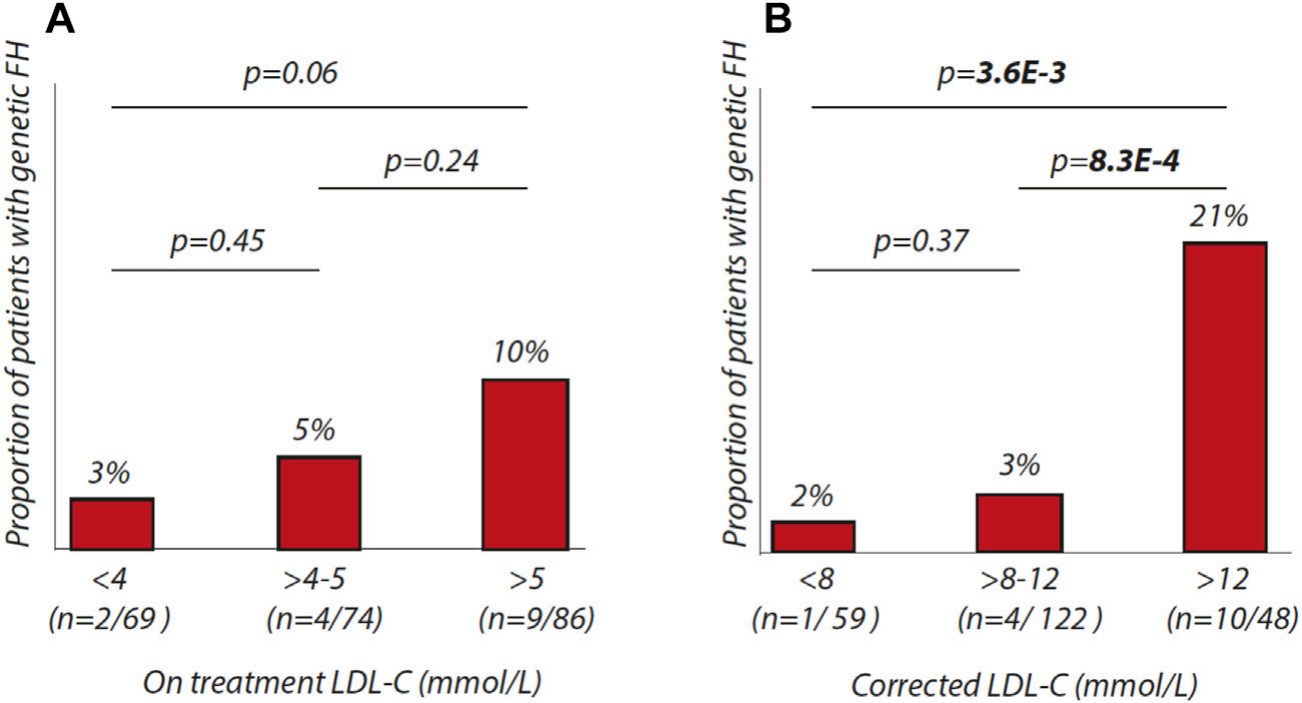
Percentages of patients with genetic FH in different LDL-C ranges. **(A)** proportion of patients with genetic FH according to on-treatment LDL-C ranges (<4 mmol/L, > 4 to 5 mmol/L, > 5 mmol/L). **(B)** proportion of patients with genetic FH according to corrected-LDL-C ranges (<8 mmol/L, > 8 to 12 mmol/L, > 12 mmol/L).

We further evaluated the proportion of patients with FH within different LDL-c ranges using on-treatment or corrected LDL-c values. We observed that the chance of identifying monogenic origins of hypercholesterolemia only tends to increase with on treatment LDL-c levels (Figure 1A). In a second step, we used LDL-c values, corrected for lipid-lowering medication with the following cut-offs: <8 mmol/L, >8 to 12 mmol/L, >12 mmol/L (Figure 1B). Here, we show that the chances of finding patients with genetic FH increase significantly over the three groups (*p* = 3.6E-3). In the group with corrected LDL-c above 12 mmol/L, 21% was found to be mutation positive.

## Discussion

The current study demonstrates that in the studied cohort of Emirati participants, we have been able to identify a genetic origin of FH in 1 out of 15 individuals with an LDL-c>95th percentile.

It is tempting to speculate that the low prevalence of FH in our study is related to the fact that these patients are characterized by not only hypercholesterolemia but also increased triglycerides and low HDL-c and recruited at the Imperial College London Diabetes Centre in Abu Dhabi, a specialised diabetes centre. In our study, participants have an average BMI of 31, fasting glucose of 8.3 mmol while 57 % were on anti-diabetic medication. Similar characteristics were seen in the Gulf FH registry (Al-Rasadi et al., 2018b). We believe, however, that it is unlikely that inclusion of patients with pre-diabetes or type 2 diabetes in our study can explain the low prevalence of genetic FH compared to studies in Western countries as insulin-resistance is generally not characterised by high LDL-c concentrations but increased levels of triglycerides and decreased levels of HDL-c (Feingold et al., 1992). On the other hand, carriers of FH causal variants are typically characterized by an isolated high LDL-c. It appears that FH causing variants in our study confer classic hypercholesterolemia on top of an unfavourable metabolic plasma lipid phenotype. This is illustrated by the fact that FH^+^ and FH^-^ patients are similar when it comes to age, BMI, blood pressure, high sensitivity CRP, plasma glucose concentration, HbA1c, HDL-c, and triglycerides plasma levels. This observation may warrant attention as patients referred to a diabetes centre can also suffer from hypercholesterolemia which likely puts them at an even higher risk of ASCVD which is especially true for identified with causal mutations in LDL genes.

Taken the lifelong genetic burden of increased LDL-c in the 15 identified FH^+^ patients combined with their detrimental metabolic phenotype; the cardiovascular consequences are anticipated to be more serious than observed in classical FH. The relatively young age of our study cohort (45 yrs), however, makes it difficult to evaluate their atherosclerotic burden and cardiac complications but it is noteworthy that ASCVD-associated mortality rate in the Middle East is one of the highest worldwide while the mean age of patients suffering from myocardial infarction in this part of the world is 10–12 years younger when compared to Western countries (Ramahi, 2010; Gehani et al., 2014).

This brings us to the FH^-^ patients for which there is no explanation for their hypercholesterolemia. Since in this study FH^+^ and FH^-^ patients have similar metabolic phenotypes, it appears unlikely that this trait would drive hypercholesterolemia in FH^-^ patients. Having excluded causal canonical gene defects, one can imagine that a better understanding of factors driving hypercholesterolemia in our FH^-^ Emirati cohort may provide novel insights into lipid metabolism. Additional studies are in this regard needed to address the origin of hypercholesterolemia in the FH^-^ patients to search for novel genes or other factors that can explain their phenotype.

Our study presents limitations. First of all, we had to account for the absence of pre-treatment LDL-c levels while 96% of our study participants were using lipid-lowering medication. Using widely used adjustment criteria for various lipid-lowering drugs (Wensel et al., 2010; Haralambos et al., 2015), we calculated pre-treatment LDL-c levels to be on average 10 mmol/l in our study cohort which we consider a solid basis to start screening for mutations in genes involved in LDL metabolism. To illustrate this point, the DLCN indicates that only LDL-c values above 8.5 mmol/l can be categorized as probable FH. This suggests that even in the case that adjustments would overestimate pre-treatment LDL-c levels, it is likely that true pre-treatment LDL-c levels are still well above 8 mmol/l. Our study also provides support that use of the corrections for LDL-c values are valid as we show that the chance of finding a mutation in LDL candidate genes increases significantly over three strata of estimated pre-treatment LDL-c (Figure 1). We were furthermore unable to retrieve information on family history and physical characteristics to complete the DLCN score. These parameters would, however, only have increased the DCLN scores which would further underline the need for molecular diagnostics in our cohort.

In conclusion, this study into the genetics of hypercholesterolemia in the selected Emirati population shows that chances of finding FH causing mutations are low (7%) when standard criteria are used, despite marked hypercholesterolemia. Taking into consideration the limitations of the study, further investigations are needed to explain the discrepancy with similar studies in Western societies. The study, however, warrants further consideration in regard to FH screening initiatives in the Gulf region and further highlights the health risks in both FH^-^ and FH^+^ individuals with hypercholesterolemia, obesity and type 2 diabetes.

## Data Availability

All datasets presented in this study are included in the article/Supplementary Material.
