# Supplementary material for: Low Detection Rates of Genetic FH in Cohort of Patients With Severe Hypercholesterolemia in the United Arabic Emirates
Source: Front Genet. 2022 Jan 3;12:809256. doi: 10.3389/fgene.2021.809256 (PMC8762259; doi:10.3389/fgene.2021.809256)
Supplement: Supplementary file 1 [file DataSheet1.PDF]

SUPPLEMENTAL

1. SUPPLEMENTAL METHODS

Targeted Next Generation Sequencing design

Our targeted Next Generation Sequencing kit was designed using the Agilent SureDesign tool (https://earray.chem.agilent.com/suredesign/). This custom Next Generation Sequencing panel consists of more than 100 genes captured for different purposes, of which 42 kb of genomic sequences are dedicated to monogenic FH genes. The four FH sequenced genes *(LDLR, APOB, PCSK9* and *LDLRAP1*) comprise 70 regions ± 50 flanking base pairs plus 2kb of *LDLR* promoter region. Multiplex amplification and library preparation were performed following the manufacturer's instructions (SureSelectQXT for custom designs from 1 kb to 499 kb). Libraries were pooled to an equimolar concentration before proceeding to 150 bp paired-end sequencing on a MiSeq sequencer (Illumina).

Bioinformatic analysis

During the first pre-processing steps of the pipeline, PhiX reads are inserted in each sample to create control SNPs in the dataset. Subsequently, Illumina encoding is checked and QC metrics are calculated using a FastQC tool. Sequence data was aligned to the human reference genome (hg19/GRCh37) using BWA-MEM (version 0.7.15) [1], resulting in a SAM file. Reads in the SAM file were sorted, converted in sorted Bam files and marked for duplicates with Sambamba [2]. Single-nucleotide variants and small indel variants were called with GATK version 3.7, using the HaplotypeCaller tool, combined in a VCF file along with additional information such as genotype quality, allele frequency, strand bias and read depth for that SNP/Indel and subsequently filtered using GATK best practices [3]. Additional annotations related to prediction scores (LRT score, MutationTaster, PolyPhen-2 HumDiv, PolyPhen-2 HumVar, and Sorting Intolerant From Tolerant [SIFT]) were added using ANNOVAR [4].

Variant filtering and assignment of FH causality

Variants predicted to affect protein coding or splicing, using SnpEFF annotations (version4.3)[5], were kept during the analysis workflow. Variants frequency was determined using the Exome Aggregation Consortium database (Exac.broadinstitute.org/ (release 0.3.1) [6], Genome of Netherlands (GoNL project http://www.nlgenome.nl) [7] and Greater Middle East Variome project (http://igm.ucsd.edu/gme/index.php) [8].

Variants were considered of interest for **autosomal dominant hypercholesterolemia** when: - their minor allele frequency **(MAF) was:**

-Lower than 0.1% in Exome Aggregation Consortium and Genome of Netherlands databases and lower than 0.5% in Greater Middle East Variome project database; - the mutation was **described in publicly available archive** of genetic variations associated with clinical phenotypes: Human Genome Mutation Database (http://www.hgmd.cf.ac.uk/[9]), FH mutation database Foundation (http://www.ucl.ac.uk/ldlr/Current/) and ClinVar (https://www.ncbi.nlm.nih.gov/clinvar [10]) ; **and/or** if the missense variant is **predicted to be deleterious** by each of 5 *in silico* prediction algorithms (LRT score, MutationTaster, PolyPhen-2 HumDiv, PolyPhen-2 HumVar and SIFT) [11].

Variants were considered of interest for **autosomal recessive hypercholesterolemia** when: - their **MAF was lower than 1%** in Exome Aggregation Consortium and Genome of Netherlands databases and lower than 2% in Greater Middle East Variome project database.

All variations reported have been validated by visual examination of sequencing reads alignment using Integrative Genome Viewer[12].

Detection of Copy Number Variations was performed using a combination of dedicated tools CoNVaDING and XHMM [13,14].

Validation of CNV

The validation of the *APOB* duplication (Table 2 – patient ID:#662), identified by NGS, was validated by using the Infinium Global Screening Array-24 v1.0-MD from Illumina (San Diego, CA 92122, https://www.illumina.com). Genotyping was performed following manufacturers' recommendations. Data were uploaded and analysed with Nexus Copy Number (https://www.biodiscovery.com/). Nexus uses log ratio for the CNV analysis. Duplications present log ratios above 0.1 and deletions have log ratios below -0.18.

SUPPLEMENTAL TABLES

Supplemental table 1: Correction factors used for on-treatment calculation of LDL-c values

Treatment (mg)	Treatment (mg)	Correction factor
Ezetimibe		
10		1,2
Pravastatin		
10		1,2
20		1,3
40		1,5
Pravastatin	Ezetimibe	
10	10	1,5
20	10	1,6
40	10	1,7
Simvastatin		
10		1,4
20		1,6
40		1,7
80		1,9
Simvastatin	Ezetemibe	
10	10	1,9
20	10	2
40	10	2,3
80	10	2,4
Atorvastatin		
10		1,6
20		1,8
40		2
80		2,2
Atorvastatin	Ezetemibe	
10	10	2
20	10	2,2
40	10	2,2
80	10	2,5
Rosuvastatin		
5		1,8
10		1,9
20		2,1
40		2,4
Rosuvastatin	Ezetimibe	
10	10	2,5
20	10	2,7
40	10	3,3
Pitavastatin		
2		1,6
4		1,8

Values are extracted from two studies [15,16]

Supplemental table 2: Rare variants identified with unknown clinical significance

Chr_POS_REF_ALT(GT)	Gene symbol	Coding variation	Protein variation	Clinical significance	Found in n patients
**Chr19_11213335_T_C(Het)**	*LDLR*	c.191-5T>C		VUS	1
**Chr19_11218079_G_A(Het)**	*LDLR*	c.829G>A	p.Glu277Lys	VUS	2
**Chr19_11218190_G_A(Het)**	*LDLR*	c.940G>A	p.Gly314Arg	VUS	1
**Chr19_11230787_A_G(Het)**	*LDLR*	c.1865A>G	p.Asp622Gly	VUS	1
**Chr19_11230903_C_T(Het)**	*LDLR*	c.1981C>T	p.Pro661Ser	VUS	1
**Chr19_11231159_G_A(Het)**	*LDLR*	c.2101G>A	p.Gly701Ser	VUS	1
**Chr19_11233940_G_A(Het)**	*LDLR*	c.2231G>A	p.Arg744Gln	VUS	1
**Chr19_11233998_G_T(Het)**	*LDLR*	c.2289G>T	p.Glu763Asp	VUS	1
**Chr19_11240240_G_A(Het)**	*LDLR*	c.2441G>A	p.Arg814Gln	VUS	1
**Chr2_21227972_A_T(Het)**	*APOB*	c.11768T>A	p.Phe3923Tyr	VUS	1
**Chr2_21228359_A_G(Het)**	*APOB*	c.11381T>C	p.Val3794Ala	VUS	1
**Chr2_21228479_G_A(Het)**	*APOB*	c.11261C>T	p.Thr3754Ile	VUS	1
**Chr2_21228960_A_G(Het)**	*APOB*	c.10780T>C	p.Trp3594Arg	VUS	1
**Chr2_21229679_G_C(Het)**	*APOB*	c.10061C>G	p.Ala3354Gly	VUS	1
**Chr2_21230498_C_T(Het)**	*APOB*	c.9242G>A	p.Ser3081Asn	VUS	1
**Chr2_21230828_T_G(Het)**	*APOB*	c.8912A>C	p.Asn2971Thr	VUS	3
**Chr2_21231058_G_C(Het)**	*APOB*	c.8682C>G	p.Asn2894Lys	VUS	1
**Chr2_21232409_C_T(Het)**	*APOB*	c.7331G>A	p.Arg2444His	VUS	1
**Chr2_21232583_A_T(Het)**	*APOB*	c.7157T>A	p.Ile2386Lys	VUS	1
**Chr2_21232811_A_G(Het)**	*APOB*	c.6929T>C	p.Ile2310Thr	VUS	1
**Chr2_21233144_G_A(Het)**	*APOB*	c.6596C>T	p.Ala2199Val	VUS	1
**Chr2_21233323_C_G(Het)**	*APOB*	c.6417G>C	p.Lys2139Asn	VUS	1
**Chr2_21234812_G_A(Het)**	*APOB*	c.4928C>T	p.Ala1643Val	VUS	1
**Chr2_21235016_T_C(Het)**	*APOB*	c.4724A>G	p.Asn1575Ser	VUS	1
**Chr2_21235303_C_A(Het)**	*APOB*	c.4437G>T	p.Leu1479Phe	VUS	1
**Chr2_21235374_C_T(Het)**	*APOB*	c.4366G>A	p.Asp1456Asn	VUS	1
**Chr2_21236085_C_T(Het)**	*APOB*	c.4163G>A	p.Arg1388His	VUS	1
**Chr2_21237422_T_C(Het)**	*APOB*	c.3740A>G	p.Tyr1247Cys	VUS	1
**Chr2_21238260_T_C(Het)**	*APOB*	c.3490A>G	p.Arg1164Gly	VUS	1
**Chr2_21239527_C_T(Het)**	*APOB*	c.3122-6G>A		VUS	1
**Chr2_21245919_A_G(Het)**	*APOB*	c.2605-5T>C		VUS	1
**Chr2_21249840_A_T(Het)**	*APOB*	c.2068-4T>A		VUS	1
**Chr2_21250786_A_C(Het)**	*APOB*	c.1981T>G	p.Phe661Val	VUS	1
**Chr2_21252855_T_C(Het)**	*APOB*	c.1385A>G	p.Glu462Gly	VUS	1
**Chr2_21260054_G_T(Het)**	*APOB*	c.611C>A	p.Ser204Tyr	VUS	1
**Chr2_21260059_T_A(Het)**	*APOB*	c.606A>T	p.Glu202Asp	VUS	1
**Chr2_21260091_C_T(Het)**	*APOB*	c.574G>A	p.Val192Ile	VUS	3
**Chr1_25889144_G_A(Het)**	*LDLRAP1*	c.469G>A	p.Val157Ile	VUS	2
**Chr1_25889633_C_A(Het)**	*LDLRAP1*	c.605C>A	p.Ser202Tyr	VUS	3
**Chr1_25891660_C_A(Het)**	*LDLRAP1*	c.748-4C>A		VUS	5
**Chr1_55505552_A_ACTGC TGCTG(Het)**	*PCSK9*	c.63_65dupGC T	p.Leu22dup	VUS	2
**Chr1_55517952_C_T(Het)**	*PCSK9*	c.525C>T	p.Asp175Asp	VUS	2
**Chr1_55518417_G_A(Het)**	*PCSK9*	c.752G>A	p.Arg251His	VUS	1
**Chr1_55524216_C_G(Het)**	*PCSK9*	c.1399C>G	p. Pro467Ala	VUS	1
**Chr1_55524222_C_T(Het)**	*PCSK9*	c.1405C>T	p.Arg469Trp	VUS	1
**Chr1_55527222_A_C(Het)**	*PCSK9*	c.1856A>C	p.Gln619Pro	VUS	1
**Chr1_55529225_C_A(Het)**	*PCSK9*	c.2047C>A	p.His683Asn	VUS	1

Legends Abbreviations

Genomic coordinates of identified variants are reported with Chromosome, Position, Reference Allele and Alternative Allele observed (Chrom_Pos_Ref_Alt) related to GRCh37 genome Human built 19. All variants listed are heterozygous. *LDLR*, Low Density Lipoprotein

Receptor (NM_000527); *APOB*, Apolipoprotein-B (NM_000384); *PCSK9*, Proprotein convertase subtilisin/kexin type 9 (NM_174936.3); *LDLRAP1*, Low Density Lipoprotein Receptor adaptor protein 1 (NM_015627); LDL-c, Low Density Lipoprotein cholesterol, M, Males; F, Females.

Supplementary Figure 1: Validation of *APOB* duplication using global screening array

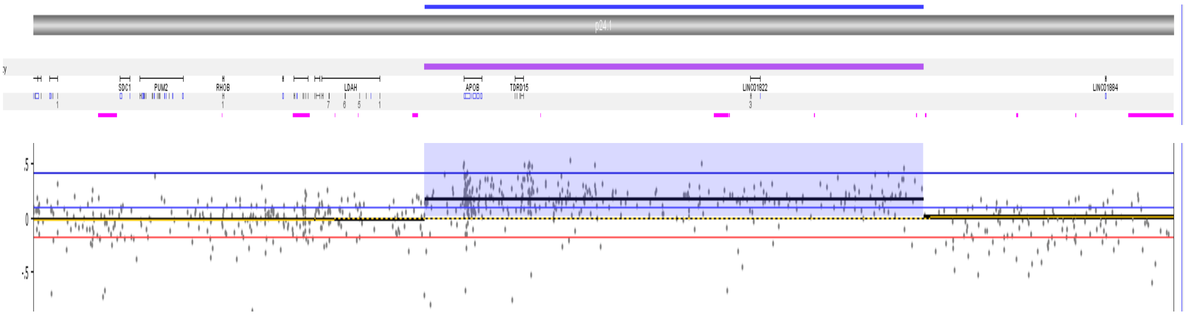

Legend:

The figure represents a screenshot, visualized in Nexus Copy Number software of the locus 2p24 for the patient #662 for who a duplication of the *APOB* gene was identified by targeted sequencing. Dots represent SNPs genotyped and Y-axis corresponds to log ratio. The lowest blue bar represents the significant threshold (log ratio above 0.1) for duplications. The red bas correspond to the threshold for deletion (log ratio below -0.18). The black bold bar correspond to the log ratio between the two alleles. The blue highlighted area corresponds to the duplication observed (coordinates chr2:21,130,084-22,324,616 Hg19) which encompasses the genes *APOB, TDRD15* and *LINC01822* (top line).

SUPPLEMENTARY REFERENCES

1. Li H, Durbin R. Fast and accurate long-read alignment with Burrows-Wheeler transform. Bioinforma Oxf Engl. 2010 Mar 1;26(5):589–95.

2. Tarasov A, Vilella AJ, Cuppen E, Nijman IJ, Prins P. Sambamba: fast processing of NGS alignment formats. Bioinforma Oxf Engl. 2015 Jun 15;31(12):2032–4.

3. Van der Auwera GA, Carneiro MO, Hartl C, Poplin R, Del Angel G, Levy-Moonshine A, Jordan T, Shakir K, Roazen D, Thibault J, Banks E, Garimella KV, Altshuler D, Gabriel S, DePristo MA. From FastQ data to high confidence variant calls: the Genome Analysis Toolkit best practices pipeline. Curr Protoc Bioinforma. 2013;43:11.10.1-33.

4. Wang K, Li M, Hakonarson H. ANNOVAR: functional annotation of genetic variants from high-throughput sequencing data. Nucleic Acids Res. 2010 Sep;38(16):e164.

5. Cingolani P, Platts A, Wang LL, Coon M, Nguyen T, Wang L, Land SJ, Lu X, Ruden DM. A program for annotating and predicting the effects of single nucleotide polymorphisms, SnpEff: SNPs in the genome of Drosophila melanogaster strain w1118; iso-2; iso-3. Fly (Austin). 2012 Jun;6(2):80–92.

6. Lek M, Karczewski KJ, Minikel EV, Samocha KE, Banks E, Fennell T, O'Donnell-Luria AH, Ware JS, Hill AJ, Cummings BB, Tukiainen T, Birnbaum DP, Kosmicki JA, Duncan LE, Estrada K, Zhao F, Zou J, Pierce-Hoffman E, Berghout J, Cooper DN, Deflaux N, DePristo M, Do R, Flannick J, Fromer M, Gauthier L, Goldstein J, Gupta N, Howrigan D, Kiezun A, Kurki MI, Moonshine AL, Natarajan P, Orozco L, Peloso GM, Poplin R, Rivas MA, Ruano-Rubio V, Rose SA, Ruderfer DM, Shakir K, Stenson PD, Stevens C, Thomas BP, Tiao G, Tusie-Luna MT, Weisburd B, Won H-H, Yu D, Altshuler DM, Ardissino D, Boehnke M, Danesh J, Donnelly S, Elosua R, Florez JC, Gabriel SB, Getz G, Glatt SJ, Hultman CM, Kathiresan S, Laakso M, McCarroll S, McCarthy MI, McGovern D, McPherson R, Neale BM, Palotie A, Purcell SM, Saleheen D, Scharf JM, Sklar P, Sullivan PF, Tuomilehto J, Tsuang MT, Watkins HC, Wilson JG, Daly MJ, MacArthur DG, Exome Aggregation Consortium. Analysis of protein-coding genetic variation in 60,706 humans. Nature. 2016 18;536(7616):285–91.

7. Genome of the Netherlands Consortium. Whole-genome sequence variation, population structure and demographic history of the Dutch population. Nat Genet. 2014 Aug;46(8):818–25.

8. Scott EM, Halees A, Itan Y, Spencer EG, He Y, Azab MA, Gabriel SB, Belkadi A, Boisson B, Abel L, Clark AG, Greater Middle East Variome Consortium, Alkuraya FS, Casanova J-L, Gleeson JG. Characterization of Greater Middle Eastern genetic variation for enhanced disease gene discovery. Nat Genet. 2016;48(9):1071–6.

9. Fokkema IFAC, den Dunnen JT, Taschner PEM. LOVD: easy creation of a locus-specific sequence variation database using an 'LSDB-in-a-box' approach. Hum Mutat. 2005 Aug;26(2):63–8.

10. Landrum MJ, Lee JM, Riley GR, Jang W, Rubinstein WS, Church DM, Maglott DR. ClinVar: public archive of relationships among sequence variation and human phenotype. Nucleic Acids Res. 2014 Jan;42(Database issue):D980-985.

11. Khera AV, Won H-H, Peloso GM, Lawson KS, Bartz TM, Deng X, van Leeuwen EM, Natarajan P, Emdin CA, Bick AG, Morrison AC, Brody JA, Gupta N, Nomura A, Kessler T, Duga S, Bis JC, van Duijn CM, Cupples LA, Psaty B, Rader DJ, Danesh J, Schunkert H, McPherson R, Farrall M, Watkins H, Lander E, Wilson JG, Correa A, Boerwinkle E, Merlini PA, Ardissino D, Saleheen D, Gabriel S, Kathiresan S. Diagnostic Yield and Clinical Utility of Sequencing Familial Hypercholesterolemia Genes in Patients With Severe Hypercholesterolemia. J Am Coll Cardiol. 2016 07;67(22):2578–89.

12. Thorvaldsdóttir H, Robinson JT, Mesirov JP. Integrative Genomics Viewer (IGV): high-performance genomics data visualization and exploration. Brief Bioinform. 2013 Mar;14(2):178–92.

13. Johansson LF, van Dijk F, de Boer EN, van Dijk-Bos KK, Jongbloed JDH, van der Hout AH, Westers H, Sinke RJ, Swertz MA, Sijmons RH, Sikkema-Raddatz B. CoNVaDING: Single Exon Variation Detection in Targeted NGS Data. Hum Mutat. 2016 May;37(5):457–64.

14. Fromer M, Purcell SM. Using XHMM Software to Detect Copy Number Variation in Whole-Exome Sequencing Data. Curr Protoc Hum Genet. 2014 Apr 24;81:7.23.1-21.

15. Wensel TM, Waldrop BA, Wensel B. Pitavastatin: a new HMG-CoA reductase inhibitor. Ann Pharmacother. 2010 Mar;44(3):507–14.

16. Haralambos K, Whatley SD, Edwards R, Gingell R, Townsend D, Ashfield-Watt P, Lansberg P, Datta DBN, McDowell IFW. Clinical experience of scoring criteria for Familial Hypercholesterolaemia (FH) genetic testing in Wales. Atherosclerosis. 2015 May;240(1):190–6.
